# CRL4^CRBN^ E3 Ligase Complex as a Therapeutic Target in Multiple Myeloma

**DOI:** 10.3390/cancers14184492

**Published:** 2022-09-16

**Authors:** Joanna Barankiewicz, Aleksander Salomon-Perzyński, Irena Misiewicz-Krzemińska, Ewa Lech-Marańda

**Affiliations:** 1 Department of Hematology, Institute of Hematology and Transfusion Medicine, 02-776 Warsaw, Poland; 2 Department of Hematology and Transfusion Medicine, Center of Postgraduate Medical Education, 02-776 Warsaw, Poland; 3 Department of Experimental Hematology, Institute of Hematology and Transfusion Medicine, 02-776 Warsaw, Poland

**Keywords:** multiple myeloma, cereblon, immunomodulatory drugs, cereblon E3 ligase modulators, proteolysis targeting chimeras

## Abstract

**Simple Summary:**

Immunomodulatory drugs (IMiDs) are effective in the treatment of multiple myeloma (MM) and other hematological malignancies. Cereblon (CRBN), a target of IMiDs, forms the CRL4 E3 ubiquitin ligase complex (CRL4^CRBN^) with DDB1, CUL4A and RBX1. The insight into the molecular mechanism of IMiDs action has advanced dramatically since the identification of cereblon (CRBN) as their direct target. Targeting CRBN by IMiDs modifies CRL4^CRBN^ substrate specificity towards non-physiological protein targets which are subsequently ubiquitinated and degraded by the proteasome. To date, IMiDs are the only known group of protein degraders used in clinical practice. This review provides the current state of knowledge about thalidomide and its derivatives’ mechanisms of action, and highlights the future perspectives for targeted protein degraders.

**Abstract:**

Multiple myeloma (MM) is the second most common hematological malignancy with a recurrent clinical course. The introduction of immunomodulatory drugs (IMiDs) was one of the milestones in MM therapy leading to a significant improvement in patients’ prognosis. Currently, IMiDs are the backbone of MM therapy in newly diagnosed and relapsed/refractory settings. It is now known that IMiDs exert their anti-myeloma activity mainly by binding cereblon (CRBN), the substrate receptor protein of the CRL4 E3 ubiquitin ligase (CRL4^CRBN^) complex. By binding CRBN, IMiDs alter its substrate specificity, leading to ubiquitination and proteasomal degradation of proteins essential for MM cell survival. Following the success of IMiDs, it is not surprising that the possibility of using the CRL4^CRBN^ complex’s activity to treat MM is being further explored. In this review, we summarize the current state of knowledge about novel players in the MM therapeutic landscape, namely the CRBN E3 ligase modulators (CELMoDs), the next generation of IMiDs with broader biological activity. In addition, we discuss a new strategy of tailored proteolysis called proteolysis targeting chimeras (PROTACs) using the CRL4^CRBN^ to degrade typically undruggable proteins, which may have relevance for the treatment of MM and other malignancies in the future.

## 1. Introduction

**Figure 1:**
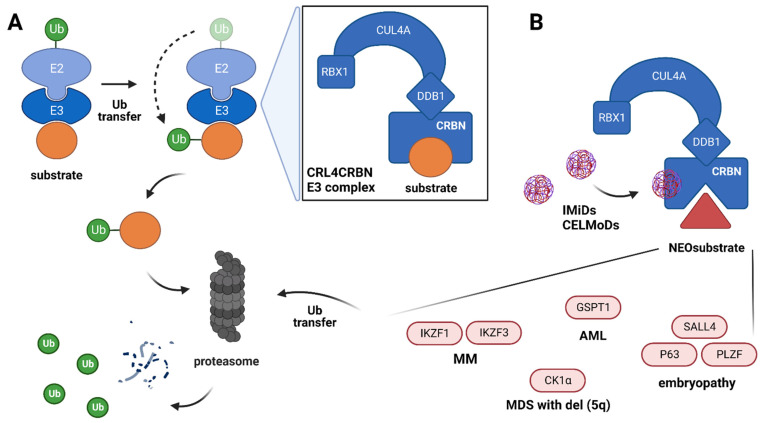
(**A**) Overview of the ubiquitination process via CRL4^CRBN^ E3 ligase complex. The E3 ligase recognizes the E2-Ub complex and target substrate with subsequent transfer of Ub from E2 to the substrate. This process results in Ub-substrate transfer to the proteasome and proteolytic degradation with Ub recycle. The CRL4^CRBN^ E3 ligase complex (enlarged) is formed by cereblon (CRBN)—a substrate recruiter, and other proteins such as DNA damage binding protein 1 (DDB1), cullin 4A (CUL4A), and regulator of cullins-1 (RBX1). (**B**) Mechanism of CRBN-mediated effects upon exposure to thalidomide and its derivatives. Binding IMiDs/CELMoDs to the CRBN leads to the recognition of different substrates (neosubstrates) for ubiquitination and successive protein degradation.

The ubiquitin-proteasome pathway plays an essential role in the proteins’ degradation. This process is mediated by a cascade of enzymatic reactions engaging a ubiquitin-activating enzyme (E1), a ubiquitin-conjugating enzyme (E2), and a ubiquitin ligase (E3), which are recycled and activated by ATP during the whole ubiquitination pathway [1]. The role of E3 is the determination of the substrate specificity for ubiquitination and subsequent degradation in the proteasome. The human genome encodes more than 600 E3 ubiquitin ligases, and the cullin-RING ubiquitin ligases (CRLs) represent the largest E3 ligase family, which take part in numerous cellular homeostatic processes, e.g., signal transduction, cell cycle regulation, DNA damage response, regulation of transcription and embryonic development [2,3]. CRL4 E3 ubiquitin ligase is a complex of RING finger domain protein (Roc1, also named RBX1), cullin4 (CUL4) scaffold protein, and DDB1–CUL4 associated proteins, which determine the substrate specificity for the CRL4 E3 activity. Cereblon (CRBN) is one of the CRL4 E3 substrate receptors, and this protein is crucial for the action of small molecules, such as immunomodulatory drugs (IMiDs). Targeting CRBN by IMiDs modifies its substrate specificity towards non-physiological proteins which are subsequently ubiquitinated and degraded by the proteasome [4,5,6,7,8,9,10], Figure 1A. This mechanism of action has shown particular relevance in the treatment of multiple myeloma (MM), the second most common hematological malignancy with a recurrent clinical course leading to 20,000 deaths per year in the European Union [11]. The introduction of IMiDs-based treatment has been a game changer for patients with MM, significantly improving their prognosis.

Although acting on the E3 ligase-related function of CRBN appears to be the main mechanism for the anti-myeloma activity of IMiDs [12,13], recent reports indicate that IMiDs also act by modulating other properties of CRBN, such as chaperone function [14,15]. Therefore, to emphasize the broader biological activity, the next generation of IMiD is called ‘CELMoDs’ (Cereblon E3 ligase modulators). In recent years, the CRL4^CRBN^ complex, along with other E3 ligases, is widely explored as the target of degradation typically “undruggable” proteins by heterobifunctional small molecules, known as proteolysis targeting chimeras (PROTACs).

Here, we review the ways of modulating CRL4^CRBN^ E3 ligase activity in a CRBN-dependent manner in established and upcoming therapeutic approaches in multiple myeloma. 

## 2. Immunomodulatory Drugs (IMiDs)

### 2.1. Mechanism of IMiDs’ Action

The introduction of thalidomide in 2006, a first-in-class IMiD, was one of the milestones in MM therapy. Together with its new generation derivatives, such as lenalidomide and pomalidomide, along with proteasome inhibitors and monoclonal antibodies, these drugs are placed as a standard of care for MM patients at all disease stages. Before the clarification of the molecular mechanism of action, thalidomide and its analogues were characterized by modulation of T cells, NK and NK-T cells functions by inducing the production of cytokines, including IL-2 (interleukin-2) and interferon-γ [16,17,18]. Thus, thalidomide and its analogs are called immunomodulatory drugs in addition to their anti-angiogenic activity, disruption of the myeloma cell-bone marrow stromal interaction, and downregulation of osteoclastogenesis [19,20].

The game-changer in the exploration of IMiDs molecular mechanism of action was information that thalidomide interacts with CRBN, and this interplay led to the teratogenic side effects and limb malformations of newborns [21]. Then, it was shown that CRBN expression was required for the anti-myeloma activity of IMiDs as CRBN knockdown leads to resistance to lenalidomide and pomalidomide in MM cell lines [12]. Our group and others showed that CRBN expression is associated with a response to thalidomide and lenalidomide-based treatment in MM patients [22,23,24,25]. Recent molecular studies with lenalidomide- and pomalidomide-resistant MM patients revealed some CRBN molecular alterations (e.g., point mutation, structural variation, copy loss, or exon 10 spliced transcript of CRBN) associated with IMiDs’ exposure [26]. Nevertheless, the low frequency and clonal fraction of identified CRBN mutations cannot be responsible for IMIDs resistance in the majority of patients [27,28]. As IMiDs resistance is one of the main challenges in MM treatment, its mechanism of resistance needs to be explored in future studies.

The subsequent key findings in IMIDs mechanism of action were presented in 2014. Two papers demonstrated that lenalidomide’s interaction with CRBN changes its substrate specificity to induce the proteasomal-dependent degradation of transcriptional factors IZKF1 and IKZF3 (named also Ikaros and Aiolos, respectively) [5,6]. IKZF1 and IKZF3 were defined as CRBN ‘neosubstrates’ because they only become CRBN targets in the presence of IMiDs. Degradation of IKZF1/3 regulates the expression of other genes, such as *IRF4* and *MYC*, and is essential for the proliferation and survival of MM cells [29,30]. Disruption of the IKZF1/3-IRF4-MYC transcriptional axis is of special importance in MM cells survival [31], in contrast to studies with primary effusion lymphoma cell lines, where IMiDs triggered downregulation of IRF4 expression independently of both IKZF1 and IKZF3 [32]. The investigation of the lenalidomide mechanism of action in other hematological malignancies, such as myelodysplastic syndrome with del5q, identified the next neosubstrate of CRBN; a casein kinase 1 α (CK1α; encoded on chromosome 5q by CSNK1A1) [9]. The deletion of the 5q region leads to reduced baseline expression of CK1α and sensitizes MDS cells to lenalidomide, which causes a unique opportunity to exert its apoptotic effect. In MM cells, inactivation of CK1α induces cell cycle arrest and overcomes the bone marrow stromal protection, indicating that lenalidomide-dependent degradation of CK1α may complement its anti-myeloma activity [33,34,35]. Moreover, the group of thalidomide neosubstrates includes also PLZF, SALL4 and P63 proteins, which were identified as its teratogenicity mediators [36,37,38,39]. Recent screening studies conducted by mass-spectrometry and high-throughput sequencing of engineered cell lines revealed the multiple potential IMiDs neosubstrates [40,41], which need to be validated under physiological conditions and translated to the clinical effects of IMiDs. The established neosubstrates for CRL4^CRBN^ E3 ligase under IMiDs impact are shown in Figure 1B. 

The differences in neosubstrates repertoire degraded under IMiDs activity may reflect the various adverse events observed during MM therapy. The most common side effect of thalidomide is chronic axonal neuropathy [42], in contrast to other IMIDs characterized by myelosuppression as the most frequent toxicity. Myelosuppressive effect of lenalidomide and pomalidomide refers to the IKZF1 degradation and subsequent downregulation of the transcription factor PU.1 [43] and GATA1 [42], resulting in neutropenia and thrombocytopenia, respectively. As thalidomide is a much less potent IKZF1 degrader relative to lenalidomide and pomalidomide, it may not induce this toxicity as strongly as its newer derivatives. 

IMiDs can modulate the CRL4^CRBN^ E3 ligase activity toward the degradation of various proteins with different affinity to the specific neosubstrates. The unique patterns of substrate specificity may translate the diversity in clinical efficacy and toxicity profile of these medicines.

### 2.2. Clinical Efficacy of IMiDs

Although the role of thalidomide in MM treatment has been steadily declining since the introduction of lenalidomide, in many countries where access to lenalidomide is limited, the combination of thalidomide, dexamethasone and bortezomib (VTD) is still the key approach in patients with newly diagnosed MM who are eligible for high-dose chemotherapy followed by autologous stem-cell transplantation (auto-HSCT). Recently, VTD induction prior to auto-HSCT has been shown to achieve an objective response (at least partial response [PR]) in almost 95% of patients, confirming previously reported results [44]. As shown in the recent phase 3 CASSIOPEIA trial, the clinical benefits of VTD in terms of depth of response, rate of measurable residual disease (MRD) negativity and progression-free survival (PFS) can be further enhanced by the addition of daratumumab, a first-in-class monoclonal antibody targeting CD38, i.e., an antigen commonly expressed on the surface of MM cells [45].

The high efficacy and favorable toxicity profile of lenalidomide have made this drug the cornerstone of most regimens currently used in MM therapy, both as initial treatment and in relapsed/refractory settings. The phase 3 PETHEMA/GEM2012 trial of 458 MM patients eligible for auto-HSCT showed significant activity of lenalidomide, dexamethasone and bortezomib (VRD) combination in pre-transplant induction (6 cycles) and post-transplant consolidation (additional 2 cycles) with high rates of both deep responses (≥very good partial response [VGPR], 75%; complete response [CR], 50%) and MRD negativity (45%) assessed after consolidation [46]. The phase 2 randomized GRIFFIN study recently showed that the addition of daratumumab to VRD induction (D-VRD) (given for 4 cycles) and post-transplant consolidation (given for additional 2 cycles) significantly improved depth of response (≥VGPR, 91% vs. 73%; ≥CR, 52% vs. 42%; MRD negativity rate, 51% vs. 20%) compared to VRD alone [47]. The efficacy and safety of D-VRD as a frontline treatment for transplant-eligible MM patients will be further evaluated in the phase 3 PERSEUS trial (NCT03710603).

The superiority of VRD over RD alone was demonstrated in the phase 3 SWOG S0777 trial in treatment-naive MM patients not intended for immediate auto-HSCT. Longer progression-free survival (PFS) (median, 43 vs. 30 months) and overall survival (OS) (median, 75 vs. 64 months) were observed in VRD compared to the RD arm [48]. The ENDURANCE trial showed that in a group of patients with no intention for immediate auto-HSCT, treatment with a combination of the second-generation proteasome inhibitor carfilzomib with RD (KRD) did not provide clinical benefit in terms of PFS over VRD [49]. More recently, the MAIA study including patients ineligible for auto-HSCT due to age or comorbidities showed that adding daratumumab to RD (DRD) led to a 47% and 32% reduction in the risk of progression and death, respectively, compared to RD alone [50]. Given these results, both VRD and DRD have been established as the preferred therapeutic options for patients with newly diagnosed MM who are not eligible for auto-HSCT [51].

The treatment of relapsed/refractory MM is a major challenge in clinical practice. For patients who have not previously been exposed to lenalidomide, RD alone (especially in frail patients) [52], or combined with carfilzomib (the ASPIRE trial) [53,54] ixazomib (the TOURMALINE trial) [55], daratumumab (the POLLUX trial) [56] or elotuzumab (the ELOQUENT-2 trial) [57,58] are highly relevant therapeutic options. In turn, for lenalidomide-refractory patients, in addition to IMID-free regimens (e.g., DKD [the CANDOR trial] [59], DVD [the CASTOR trial] [60] and KD alone [the ENDEAVOR trial] [61], pomalidomide-based approaches are of great clinical value. Depending on previous therapies, performance status and comorbidities, patients with relapsed/refractory MM may benefit from pomalidomide-dexamethasone given alone or in combination with anti-CD38 antibodies (i.e., daratumumab (the APOLLO trial) [62] or isatuximab [the ICARIA-MM trial] [63], elotuzumab (the ELOQUENT-3 trial) [64], proteasome inhibitors (i.e., bortezomib [the OPTIMISMM trial] [65], carfilzomib [66] and ixazomib [67]) along with cytotoxic agents (e.g., cyclophosphamide [68]). The results of the randomized clinical trials with IMiDs are summarized in Table 1. 

Lenalidomide is also placed as the standard of care in maintenance therapy of MM after auto-HSCT or in nontransplant settings for newly diagnosed patients. In four phase 3 randomized trials, prolonged PFS was observed with hazard ratios (HRs) ranging from 0.47 to 0.57 in favor of the lenalidomide arm vs observation/placebo post auto-HSCT [69,70,71,72]. Moreover, three clinical trials’ meta-analysis documented longer overall survival (OS) of patients with lenalidomide maintenance [73]. On the other hand, one is aware of the risk of secondary malignancies during long-term exposure to lenalidomide [74], especially the several recent reports that emerged about acute B-cell leukemia with diverse clinical courses and treatment outcomes [75,76,77,78]. 

There is room for pomalidomide and new cereblon E3 ligase modulators (CELMoDs, described below) in the maintenance therapy of MM because of their higher efficacy and more favorable toxicity profile, which is of special interest during long-term therapy.

**Table 1:** Summarize the results of randomized clinical trials with IMiDs.

Trial	Phase	Regimen	Outcome
**Newly-Diagnosed MM with Transplant Intent**			
CASSIOPEIA [45]	3	Dara-VTD VTD	mPFS: NR vs. NR (HR = 0.47; *p* < 0.0001) MRD (-): 64% vs. 44% (*p* < 0.0001)
PETHEMA/GEM2012 [46]	3	VRD	mPFS: NR; MRD (-): 29% (post induction), 42% (post auto-HSCT) and 45% (post consolidation)
GRIFFIN [47]	3	Dara-VRD VRD	2y-PFS: 96% vs. 90% MRD (-): 51% vs. 20% (*p* < 0.0001)
**Newly-Diagnosed MM with Non-Transplant Intent**			
SWOG S0777 [48]	3	VRD RD	mPFS: 43 vs. 30 mo (HR = 0.71; *p* = 0.0018) mOS: 75 vs. 64 mo (HR = 0.71; *p* = 0.025)
ENDURANCE [49]	3	KRD VRD	mPFS: 34.6 vs. 34.4 months (*p* = 0.74)
MAIA [50]	3	Dara-RD VRD	mPFS: NR vs. 34.4 mo (HR = 0.53; *p* < 0.0001)
**Relapsed/Refractory MM**			
Dimopoulos et al. [52]	3	RD placebo-D	mTTP, 11.3 vs. 4.7 months (*p* < 0.001)
ASPIRE [54]	3	KRD RD	mPFS: 26 vs. 18 mo (HR 0.69; *p* = 0.0001) mOS: 48 vs. 40 mo (HR = 0.79; *p* = 0.0045)
TOURMALINE [55]	3	IRD placebo-RD	mPFS: 20.6 vs. 14.7 mo (HR = 0.74; *p* = 0.01)
POLLUX [56]	3	Dara-RD RD	mPFS: 44.5 vs. 17.5 mo (HR = 0.44; *p* < 0.0001)
ELOQUENT-2 [57,58]	3	Elo-RD RD	mPFS: 19.4 vs. 14.9 mo (HR = 0.70; *p* < 0.001) mOS: 48.3 vs. 39.6 mo (HR = 0.82; *p* = 0.04)
CANDOR [59]	3	Dara-KD KD	mPFS: 28.6 vs. 15.2 mo (HR = 0.59; *p* <0.0001)
CASTOR [60]	3	Dara-VD VD	mPFS: 16.7 vs. 7.1 mo (HR = 0.31; *p* < 0.0001)
ENDEAVOR [61]	3	KD VD	mPFS: 18.7 vs. 9.4 mo (HR = 0.53; *p* < 0.0001) mOS, 47.6 vs. 40 mo (HR = 0.79; *p* = 0.01)
APOLLO [62]	3	Dara-PD PD	mPFS: 12.4 vs. 6.9 mo (HR = 0.63; *p* = 0.0018)
ICARIA-MM [63]	3	Isa-PD PD	mPFS: 11.5 vs. 6.5 mo (HR = 0.596; *p* = 0.001) mOS: 24.6 vs. 17.7 mo (HR = 0.76; *p* = 0.028)
ELOQUENT-3 [64]	2	Elo-PD PD	mPFS: 10.3 vs. 4.7 mo (HR = 0.54; *p* = 0.008) mOS: 29.8 vs. 17.4 mo (HR = 0.59; *p* = 0.0217)
OPTIMISMM [65]	3	PVD VD	mPFS: 11.2 vs. 7.1 mo (HR = 0.61; *p* < 0.0001)

**Abbreviations:** D: dexamethasone; Dara: daratumumab; Elo: elotuzumab; HR: hazard ratio; IRD: ixazomib, lenalidomide and dexamethasone; Isa: isatuximab; KD: carfilzomib and dexamethasone; KPD: carfilzomib, pomalidomide and dexamethasone; KRD: carfilzomib, lenalidomide and dexamethasone; MM: multiple myeloma; MRD: measurable residual disease; mo: months; mOS: median OS; mPFS: median PFS; NR: not reached; ORR: objective response rate; OS: overall survival; PD: pomalidomide and dexamethasone, PFS: progression-free survival; PVD: bortezomib, pomalidomide and dexamethasone; RD: lenalidomide and dexamethasone; TTP: time to progression; VD: bortezomib and dexamethasone; VRD: bortezomib, lenalidomide and dexamethasone; VTD: bortezomib, thalidomide and dexamethasone; y, years.

## 3. Cereblon E3 Ligase Modulators (CELMoDs)

### 3.1. Mechanism of CELMoDs’ Action

**Figure 2:**
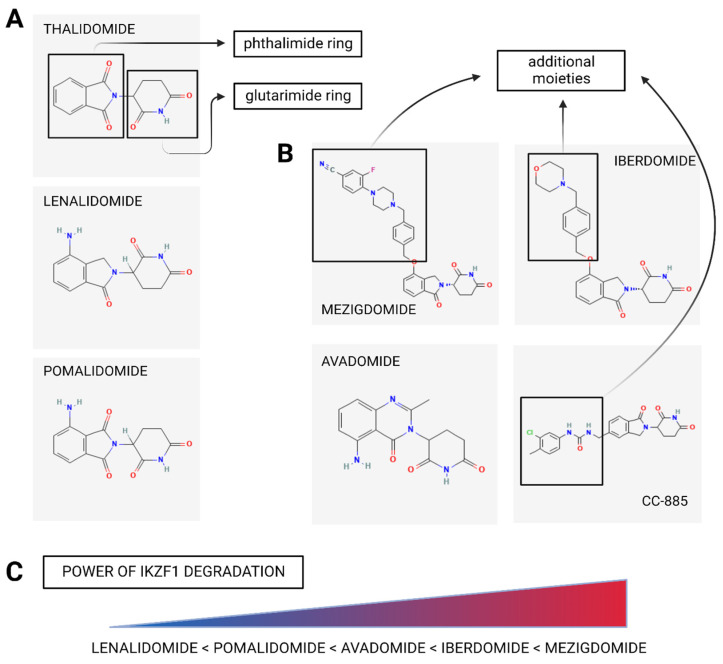
IMIDs/CELMoDs differences in chemical structure and power of IKZF1 degradation. Chemically, IMiDs (**A**) and CELMoDs (**B**) share glutarimide ring for binding to the tri-tryptophan pocket of CRBN, but the second structural region varies between each drug and determines the interaction with CRBN and neoubstrates. (**C**) The comparison of the IMiDs and CELMoDs potency in IKZF1 degradation.

Even though the enigma of IMiDs’ different ways of action is still not fully deciphered, we have to make room for the novel, intentionally designed, class of CRL4^CRBN^ players, referred to as CRBN E3 ligase modulation drugs (CELMoDs). This group of “next-generation” IMiDs is represented by CC-92480 (mezigdomide), CC-220 (iberdomide), CC-122 (avadomide) and CC-885. Chemically, CELMoDs share with IMiDs the conserved glutarimide rings for interaction with CRBN. The second, extended region of their structures (corresponding to the phthalimide ring in thalidomide) varies between each CELMoD and determines the interaction with CRBN and new CRL4^CRBN^ E3 substrates, as shown in Figure 2A,B.

One of the key features that differentiate CELMoDs from IMiDs is the enhanced affinity to the CRBN. The raw data varies between the published results depending on the used assays. Still, most publications document the approximately 10–20-fold higher CRBN-affinity of CELMoDs compared to lenalidomide or pomalidomide [79,80]. Consistent with increased affinity, the greater CELMoDs’ potency in degradation of IKZF1 and IKZF3 is observed compared to classical IMiDs [81], Figure 2C.

In contrast to known IMIDs’ neosubstrates, CC-885 was found to exert the antitumor activity by CRBN-dependent ubiquitination and degradation of the translational terminal factor, GSPT1. Degradation of GSPT1 is detrimental in acute myeloid leukemia (AML) cell lines and patient-derived AML samples [82]. Furthermore, a recent analysis based on mass-spectroscopy proteomics also identified dose- and time-dependent degradation of BNIP3L in CRBN^+/+^, but not CRBN^−/−^ cells exposed to CC-885 compound. That data uncover a novel role of CC-885 in regulating degradation of mitochondria (mitophagy) by targeting BNIP3L for CRL4^CRBN^ E3 ligase-dependent ubiquitination [83]. In MM cell lines, CC-885 selectively induced the ubiquitination and degradation of CDK4 in a CRBN-dependent manner, suggesting that CDK4 destruction contributed to its cytotoxicity in MM pre-clinical model [84].

The CC-220 has a higher than IMiDs affinity to CRBN and potency for IKZF1/3 degradation, but does not degrade CK1a or GSPT1. It is worth mentioning that iberdomide and other CELMoDs (CC-122, CC-92480) have the activity in lenalidomide- or pomalidomide-resistant cell lines with decreased CRBN expression [85,86].

Unlike other IMiDs or CELMoDs, recent basic studies with avadomide revealed the CRBN-dependent degradation of ZMYM2 (ZNF198), a transcriptional factor involved in rearrangements with FGFR1 and FLT3. This makes CC-122 a potential drug for patients with aggressive hematological malignances harboring translocations resulting in fusion oncoproteins ZMYM2–FGFR1 and ZMYM2–FLT3 [87].

### 3.2. Clinical Efficacy of CELMoDs

#### 3.2.1. CC-92480 (Mezigdomide)

Recently, the preliminary results of the phase 1/2 CC-92480-MM-002 study have been reported. A total of 19 patients with relapsed/refractory MM after a median of 3 (range, 2–4) lines of prior therapy had received a combination of CC-92480 (mezigdomide), bortezomib and dexamethasone [88]. All patients were previously exposed to lenalidomide and half of them received pomalidomide in addition. The mezigdomide-bortezomib-dexamethasone combination has shown promising clinical activity with an objective response (≥PR) achieved in almost 75% of cases and a median duration of response of 10 months. The toxicity profile was predictable and acceptable, with cytopenias being the most commonly reported grade 3 or 4 treatment-emergent adverse event. In this study, evaluation of other mezigdomide-dexamethasone combinations containing a next-generation PI (carfilzomib or ixazomib) or anti-CD38 antibody (daratumumab or isatuximab) or anti-SLAMF7 antibody (elotuzumab) is planned (NCT03989414). Another phase 1 study (NCT03374085) has recently demonstrated the mezigdomide-dexamethasone doublet to be an effective approach in a group of 66 heavily pre-treated (a median of 6 previous therapies) patients with prior exposure to lenalidomide (89%), pomalidomide (83%) and anti-CD38 antibodies (78%) [89]. The objective response rate at the therapeutic dose was almost 50%, and responses were achieved independently of resistance to IMIDs. The most common adverse events were myelosuppression. The study is ongoing, and further findings are highly anticipated.

#### 3.2.2. Iberdomide (CC-220)

Triplet combinations with iberdomide (CC-220) have shown a favorable safety profile and promising clinical activity in heavily pretreated MM patients, according to the preliminary results of the phase 1/2 CC-220-MM-001 study (NCT02773030) [90]. The iberdomid-daratumumab-dexamethasone (IberDd) cohort included 63% and 58% of daratumumab-resistant and quadruple-refractory (defined as refractory to ≥1 IMIDs, 1 PI, 1 anti-CD38 monoclonal antibodies and 1 steroid) patients, respectively. Similarly, a high representation of refractory patients (PI-refractory, 76%; quadruple-refractory, 48%) was included in the iberdomide-bortezomib-dexamethasone (IberVD) cohort. Nevertheless, the objective response rate was 35% in the IberDd and 50% in the IberVD cohort. Importantly, responses to IberDd and IberVd were achieved irrespective of daratumumab- and bortezomibe-refractoriness. It is worth highlighting that a significant proportion of patients derived clinical benefit from iberdomide-based therapy due to achieving a minimal response or stable disease. The clinical benefit rate and the disease control rate were 47% and 88% (for the IberDd cohort) and 65% and 85% (for IberVD cohort), respectively [90]. Cytopenias were the most common complication of the combination therapy. The IberDd combination for the treatment of relapsed/refractory MM is planned to be compared with DRd in the phase 3 EXCALIBER-RRMM trial (NCT04975997). Additionally, IberVD as a frontline approach for MM patients ineligible for HDT-auto-HSCT will be evaluated in the phase 2 BOREALIS trial (NCT05272826).

In the phase 1/2 CC-220-MM-001 study (NCT02773030), iberdomide in combination with dexamethasone was evaluated [91]. Almost all of the 107 enrolled patients were triple refractory (refractory to IMID, PI and anti-CD38 monoclonal antibody), 25% had an extramedullary disease, and 30% had high-risk cytogenetics. Treatment with iberdomide and dexamethasone led to a response in 26% of patients. Median PFS and OS were 3 and 11 months, respectively. Interestingly, patients who had previously received anti-BCMA therapy had similar response rates (ORR of 25%). There were no new concerns about the toxicity of the combination therapy. The efficacy and safety of iberdomid-dexamethasone combined with other anti-myeloma agents, i.e., carfilzomib (NCT05199311, NCT02773030), ixazomib (NCT04998786), cyclophosphamide (NCT04392037) and idecabtagene vicleucel (the KarMMa-7 trial, NCT04855136) are currently being investigated in several early phase studies.

#### 3.2.3. Avadomide (CC-122)

The results of the first-in-human study of avadomide monotherapy in the treatment of various advanced hematological malignancies, including two cases of heavily pretreated MM have recently been published [92]. Although no objective responses were observed, in one MM case avadomide led to long-term disease stabilization. In two other early phase studies, avadomide both in monotherapy and in combination with the anti-CD20 antibodies showed promising efficacy in the treatment of relapsed/refractory non-Hodgkin’s lymphoma [93,94]. 

#### 3.2.4. CC-885

Another CELMoD, CC-885 has shown anti-cancer activity in several preclinical studies [83,84,95]. However, to the best of our knowledge, CC-885 is not yet evaluated in clinical trials.

## 4. Proteolysis Targeting Chimeras (PROTACs)

**Figure 3:**
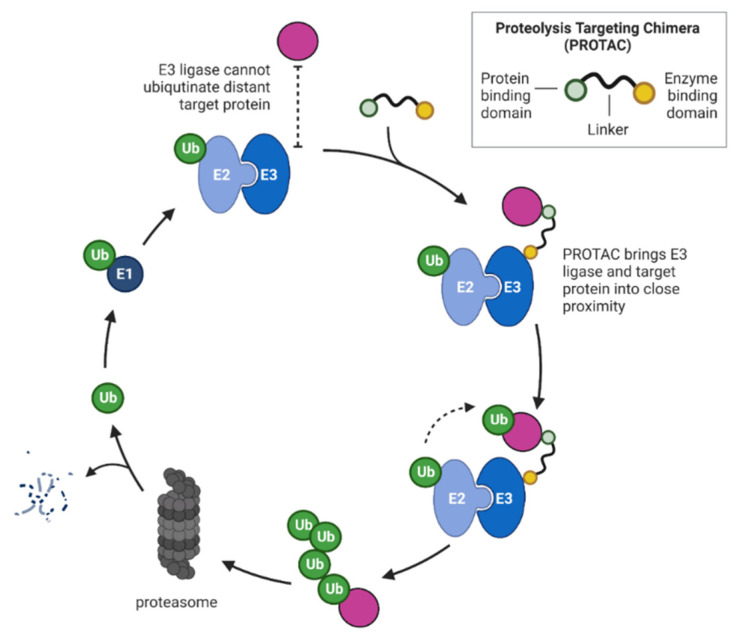
The mechanism of PROTAC-mediated targeted protein degradation. The PROTAC works as “molecular glue” and brings the target protein closer to E3 ligase to form a multi-protein complex. This result in Ub transfer from E2 to the target and subsequent degradation in the proteasome. Excluding the proteolyzed target, the classic component of the ubiquitination pathway and PROTAC recycle during the whole process.

As described previously, selective protein degradation is a treatment strategy of high clinical value, and this therapeutic approach is desirable not only for MM patients. An interesting method for novel drug design is to hijack the activity of E3 ubiquitin ligases for ubiquitination and degradation of the proteins of “our” interest (POIs). The extensive studies in IMiDs mechanism of action led to the development of “degronimids”—bifunctional compounds in which a thalidomide-like element is paired with one of many different small molecules to cause ubiquitination of proteins binding to these latter molecules [96]. This engineered technique for protein degradation is more commonly known as proteolysis targeting chimeras (PROTACs). The PROTAC molecules consist of three elements: (1) a small molecule compound that binds specifically to the target protein, (2) a compound that binds specifically to the E3 ubiquitin ligase, often called “molecular glue” and (3) a “linker” that connects the two above elements and also affects its tertiary structure, water solubility, and stability, Figure 3. 

The PROTAC technique does not require binding to the target protein’s active site, so this approach has a great advantage in overcoming the potential limitations of classical small-molecule protein inhibitors (transient targeting of non-covalent inhibitors; resistance caused by protein overexpression or point mutations). This novel strategy brings us closer to degrading “undruggable” proteins, such as crucial oncogenic proteins.

Currently, most PROTACs use the CRL4^CRBN^ and von Hippel-Lindau (VHL) E3 ubiquitin ligase as a recruiting ligase. Thus, IMiDs are often considered pioneers in respect to the “molecular glue” part of PROTAC since they promote the interaction of CRBN with a multitude of therapeutically relevant neosubstrates. 

The first CRBN-based PROTAC was developed in 2015, with the structure of thalidomide capturing CRBN and bromodomains as protein of interest (by BET inhibitor—JQ1). The resulting compound dBET1 has been shown to induce highly selective CRBN-dependent BET protein degradation in MM and AML cell lines [96]. The next-generation PROTACs based on CRL4^CRBN^—pomalidomide interaction also targets BET proteins (ARV 825), which showed promising activity against MM cells, including in vivo activity in a mice model [97,98]. Effective PROTACs targeting other MM promising oncoproteins such as CDK4 and CDK6 [99,100] and MCL-1 [101] have also been described.

It should be noted that in the case of MM, this strategy may be limited due to the resistance of MM cells that arises during treatment with IMiDs or CELMoDs. This may affect the efficacy of PROTACs based on the CRL4^CRBN^ E3 ubiquitin ligase by changes in the CRBN expression and mutation in the gene encoding CRBN. Fortunately, the human genome encodes more than 600 E3 ubiquitin ligases [3,102], so far only a few have been used for PROTAC’s generation: VHL, MDM2 (Murine double minute 2), IAPs (inhibitor of apoptosis proteins) and CRBN. The latest comprehensive investigation of PROTACs targeting different proteins but running via the same E3 ligase showed cross-resistance. In turn, the sequential exposure to other E3 ligases (CRBN of VHL) for the same target overcame this effect [103].

To date, degronimids and other PROTACs are being studied extensively in a broad spectrum of hematologic malignancies and other cancers in preclinical studies [104,105,106]. In 2019, the first potential PROTAC-based drugs entered the first-in-human clinical trial in metastatic and castration-resistant prostate cancer (ARV-110; NCT03888612) and advanced breast cancer (ARV-471; NCT04072952), resulting in acceptable toxicity profile and the first evidence of the PROTACs’ clinical activity [107,108]. In August 2022, clinical trials with ARV-110 (ADRENT, NCT0388861) and ARV-471 (VERITAC, NCT04072952) are running phase 2 trials. In the hematology field, a first-in-human phase 1 trial of a first-in-class oral BTK degrader with IMiD-like activity (NX-2127), is currently enrolling the patients with relapsed/refractory B-cell malignancies (NCT04830137). Similarly, another BTK degrader with IMID backbone (NX-5948) has entered the 1 phase trial in adults with relapsed/refractory B-cell malignancies, including also primary central nervous system lymphoma (NCT05131022). Recently, the STAT3 degrader (KT-333) also on the IMiD backbone was approved to enter the phase 1 trial in adults with refractory B-cell non-Hodgkin lymphoma, T-cell lymphomas and solid tumors [109].

## 5. Conclusions and Future Directions

The introduction of IMiDs has changed the therapeutic landscape of multiple myeloma once and for all, and together with other advances, has led to significant improvement in MM treatment outcomes. Currently, these drugs are the standard of care for induction therapy for newly-diagnosed MM patients, maintenance therapy after auto-HSCT, and treatment of relapsed/refractory MM. 

The lifetime of thalidomide from a teratogenic “dark remedy” to the first-in-class IMiD, along with an extensive investigation of its mechanism of action, gave us a unique lesson about the possibility of precise and re-directed protein ubiquitination. The identification of CRBN as a thalidomide binding protein was followed by the discovery that IMiDs modulate the ubiquitin ligase activity of CRL4^CRBN^ towards non-physiological targets for proteasome degradation. For now, plenty of new CRL4^CRBN^ interactors have been discovered as a result of broad IMiDs/CELMoDs activity investigations. The design and development of selective protein degraders based on CRL4^CRBN^ and other E3 ligases may represent the quintessence of personalized medicine, as targeted protein degraders apparently can induce degradation of any cancer vulnerability.

CELMoDs seem to be an attractive therapeutic option for MM refractory to IMiDs, but further deep proteomic investigations of resistant MM cells (especially at the stage of MRD) can reveal resistance mediating “undruggable” proteins that can become targets for PROTACs utility. 

Even though IMiDs are one of the most important drugs used in MM therapy, the landscape of their therapeutic area is enlarging to other hematologic malignancies. The efficacy of lenalidomide was proven in the treatment of relapsed mantle cell lymphoma, follicular lymphoma and marginal zone lymphomas. Furthermore, CELMoDs recruitment of new CRL4^CRBN^ substrates (e.g., GSTP1) makes them attractive for the treatment of AML. The pluripotent mechanism of IMiDs/CELMoDs action makes them attractive to complement other therapies, especially immunotherapy. The potential to enhance anti-tumor immune responses by overcoming an immunosuppressive effect of the tumor microenvironment brings them promising candidates for combined therapies with immune-engagers, such as monoclonal or bispecific antibodies and chimeric antigen receptor (CAR)-T cell therapies. 

## References

[1] Finley D., Ciechanover A., Varshavsky A. (2004). Ubiquitin as a central cellular regulator. Cell.

[2] Nguyen H.C., Wang W., Xiong Y. (2017). Cullin-RING E3 Ubiquitin Ligases: Bridges to Destruction. Macromol. Protein Complexes.

[3] Nguyen V.-N., Huang K.-Y., Weng J.T.-Y., Lai K.R., Lee T.-Y. (2016). UbiNet: An online resource for exploring the functional associations and regulatory networks of protein ubiquitylation. Database.

[4] Zhu Y.X., Braggio E., Shi C.-X., Kortuem K.M., Bruins L.A., Schmidt J.E., Chang X.-B., Langlais P., Luo M., Jedlowski P. (2014). Identification of cereblon-binding proteins and relationship with response and survival after IMiDs in multiple myeloma. Blood.

[5] Lu G., Middleton R.E., Sun H., Naniong M., Ott C.J., Mitsiades C.S., Wong K.-K., Bradner J.E., Kaelin W.G. (2014). The Myeloma Drug Lenalidomide Promotes the Cereblon-Dependent Destruction of Ikaros Proteins. Science.

[6] Krönke J., Udeshi N.D., Narla A., Grauman P., Hurst S.N., McConkey M., Svinkina T., Heckl D., Comer E., Li X. (2014). Lenalidomide Causes Selective Degradation of IKZF1 and IKZF3 in Multiple Myeloma Cells. Science.

[7] Gandhi A.K., Kang J., Havens C.G., Conklin T., Ning Y., Wu L., Ito T., Ando H., Waldman M.F., Thakurta A. (2013). Immunomodulatory agents lenalidomide and pomalidomide co-stimulate T cells by inducing degradation of T cell repressors I karos and A iolos via modulation of the E 3 ubiquitin ligase complex CRL 4^CRBN^. Br. J. Haematol..

[8] Fischer E.S., Böhm K., Lydeard J.R., Yang H., Stadler M.B., Cavadini S., Nagel J., Serluca F., Acker V., Lingaraju G.M. (2014). Structure of the DDB1–CRBN E3 ubiquitin ligase in complex with thalidomide. Nature.

[9] Krönke J., Fink E.C., Hollenbach P.W., Macbeth K.J., Hurst S.N., Udeshi N.D., Chamberlain P.P., Mani D.R., Man H.W., Gandhi A.K. (2015). Lenalidomide induces ubiquitination and degradation of CK1α in del(5q) MDS. Nature.

[10] Petzold G., Fischer E.S., Thomä G.P.E.S.F.N.H. (2016). Structural basis of lenalidomide-induced CK1α degradation by the CRL4CRBN ubiquitin ligase. Nature.

[11] Siegel R.L., Miller K.D., Jemal A. (2019). Cancer statistics, 2019. CA Cancer J. Clin..

[12] Zhu Y.X., Braggio E., Shi C.-X., Bruins L.A., Schmidt J.E., Van Wier S., Chang X.-B., Bjorklund C.C., Fonseca R., Bergsagel P.L. (2011). Cereblon expression is required for the antimyeloma activity of lenalidomide and pomalidomide. Blood.

[13] Franssen L.E., Nijhof I.S., Couto S., Levin M.-D., Bos G.M., Broijl A., Klein S.K., Ren Y., Wang M., Koene H.R. (2018). Cereblon loss and up-regulation of c-Myc are associated with lenalidomide resistance in multiple myeloma patients. Haematologica.

[14] Heider M., Eichner R., Stroh J., Morath V., Kuisl A., Zecha J., Lawatscheck J., Baek K., Garz A.-K., Rudelius M. (2021). The IMiD target CRBN determines HSP90 activity toward transmembrane proteins essential in multiple myeloma. Mol. Cell.

[15] Costacurta M., He J., Thompson P.E., Shortt J. (2021). Molecular Mechanisms of Cereblon-Interacting Small Molecules in Multiple Myeloma Therapy. J. Pers. Med..

[16] Haslett P.A., Corral L.G., Albert M., Kaplan G. (1998). Thalidomide Costimulates Primary Human T Lymphocytes, Preferentially Inducing Proliferation, Cytokine Production, and Cytotoxic Responses in the CD8+ Subset. J. Exp. Med..

[17] Davies F., Raje N., Hideshima T., Lentzsch S., Young G., Tai Y.-T., Lin B., Podar K., Gupta D., Chauhan D. (2001). Thalidomide and immunomodulatory derivatives augment natural killer cell cytotoxicity in multiple myeloma. Blood.

[18] Hayashi T., Hideshima T., Akiyama M., Podar K., Yasui H., Raje N., Kumar S., Chauhan D., Treon S.P., Richardson P. (2004). Molecular mechanisms whereby immunomodulatory drugs activate natural killer cells: Clinical application. Br. J. Haematol..

[19] D’Amato R.J., Loughnan M.S., Flynn E., Folkman J. (1994). Thalidomide is an inhibitor of angiogenesis. Proc. Natl. Acad. Sci. USA.

[20] Charliński G., Vesole D.H., Jurczyszyn A. (2021). Rapid Progress in the Use of Immunomodulatory Drugs and Cereblon E3 Ligase Modulators in the Treatment of Multiple Myeloma. Cancers.

[21] Ito T., Ando H., Suzuki T., Ogura T., Hotta K., Imamura Y., Yamaguchi Y., Handa H. (2010). Identification of a Primary Target of Thalidomide Teratogenicity. Science.

[22] Broyl A., Kuiper R., Van Duin M., Van Der Holt B., El Jarari L., Bertsch U., Zweegman S., Buijs A., Hose D., Lokhorst H.M. (2013). High cereblon expression is associated with better survival in patients with newly diagnosed multiple myeloma treated with thalidomide maintenance. Blood.

[23] Barankiewicz J., Szumera-Ciećkiewicz A., Salomon-Perzyński A., Wieszczy P., Malenda A., Garbicz F., Prochorec-Sobieszek M., Misiewicz-Krzemińska I., Juszczyński P., Lech-Marańda E. (2021). The CRBN, CUL4A and DDB1 Expression Predicts the Response to Immunomodulatory Drugs and Survival of Multiple Myeloma Patients. J. Clin. Med..

[24] Heintel D., Rocci A., Ludwig H., Bolomsky A., Caltagirone S., Schreder M., Pfeifer S., Gisslinger H., Zojer N., Jäger U. (2013). High expression of cereblon (*CRBN*) is associated with improved clinical response in patients with multiple myeloma treated with lenalidomide and dexamethasone. Br. J. Haematol..

[25] Misiewicz-Krzeminska I., De Ramón C., Corchete L.A., Krzeminski P., Rojas E.A., Isidro I., García-Sanz R., Martínez-López J., Oriol A., Bladé J. (2020). Quantitative expression of Ikaros, IRF4, and PSMD10 proteins predicts survival in VRD-treated patients with multiple myeloma. Blood Adv..

[26] Gooding S., Ansari-Pour N., Towfic F., Estévez M.O., Chamberlain P.P., Tsai K.-T., Flynt E., Hirst M., Rozelle D., Dhiman P. (2021). Multiple cereblon genetic changes are associated with acquired resistance to lenalidomide or pomalidomide in multiple myeloma. Blood.

[27] Jones J.R., Barber A., Le Bihan Y.-V., Weinhold N., Ashby C., Walker B.A., Wardell C.P., Wang H., Kaiser M.F., Jackson G.H. (2021). Mutations in CRBN and other cereblon pathway genes are infrequently associated with acquired resistance to immunomodulatory drugs. Leukemia.

[28] Salomon-Perzyński A., Barankiewicz J., Machnicki M., Misiewicz-Krzemińska I., Pawlak M., Radomska S., Krzywdzińska A., Bluszcz A., Stawiński P., Rydzanicz M. (2022). Tracking Clonal Evolution of Multiple Myeloma Using Targeted Next-Generation DNA Sequencing. Biomedicines.

[29] Shaffer A.L., Emre N.C.T., Lamy L., Ngo V.N., Wright G., Xiao W., Powell J., Dave S., Yu X., Zhao H. (2008). IRF4 addiction in multiple myeloma. Nature.

[30] Lopez-Girona A., Heintel D., Zhang L.-H., Mendy D., Gaidarova S., Brady H., Bartlett J.B., Schafer P.H., Schreder M., Bolomsky A. (2011). Lenalidomide downregulates the cell survival factor, interferon regulatory factor-4, providing a potential mechanistic link for predicting response. Br. J. Haematol..

[31] Bjorklund C.C., Lu L., Kang J., Hagner P., Havens C.G., Amatangelo M., Wang M., Ren Y., Couto S.S., Breider M. (2015). Rate of CRL4CRBN substrate Ikaros and Aiolos degradation underlies differential activity of lenalidomide and pomalidomide in multiple myeloma cells by regulation of c-Myc and IRF4. Blood Cancer J..

[32] Patil A., Manzano M., Gottwein E. (2018). CK1α and IRF4 are essential and independent effectors of immunomodulatory drugs in primary effusion lymphoma. Blood.

[33] Manni S., Carrino M., Manzoni M., Gianesin K., Nunes S.C., Costacurta M., Tubi L.Q., Macaccaro P., Taiana E., Cabrelle A. (2017). Inactivation of CK1α in multiple myeloma empowers drug cytotoxicity by affecting AKT and β-catenin survival signaling pathways. Oncotarget.

[34] Carrino M., Tubi L.Q., Fregnani A., Nunes S.C., Barilà G., Trentin L., Zambello R., Semenzato G., Manni S., Piazza F. (2019). Prosurvival autophagy is regulated by protein kinase CK1 alpha in multiple myeloma. Cell Death Discov..

[35] Hu Y., Song W., Cirstea D., Lu D., Munshi N.C., Anderson K.C. (2014). CSNK1α1 mediates malignant plasma cell survival. Leukemia.

[36] Matyskiela M.E., Couto S., Zheng X., Lu G., Hui J., Stamp K., Drew C., Ren Y., Wang M., Carpenter A. (2018). SALL4 mediates teratogenicity as a thalidomide-dependent cereblon substrate. Nat. Chem. Biol..

[37] A Donovan K., An J., Nowak R.P., Yuan J.C., Fink E.C., Berry B.C., Ebert B.L., Fischer E.S. (2018). Thalidomide promotes degradation of SALL4, a transcription factor implicated in Duane Radial Ray syndrome. eLife.

[38] Asatsuma-Okumura T., Ando H., De Simone M., Yamamoto J., Sato T., Shimizu N., Asakawa K., Yamaguchi Y., Ito T., Guerrini L. (2019). p63 is a cereblon substrate involved in thalidomide teratogenicity. Nat. Chem. Biol..

[39] Yamanaka S., Murai H., Saito D., Abe G., Tokunaga E., Iwasaki T., Takahashi H., Takeda H., Suzuki T., Shibata N. (2021). Thalidomide and its metabolite 5-hydroxythalidomide induce teratogenicity via the cereblon neosubstrate PLZF. EMBO J..

[40] An J., Ponthier C.M., Sack R., Seebacher J., Stadler M.B., Donovan K., Fischer E.S. (2017). pSILAC mass spectrometry reveals ZFP91 as IMiD-dependent substrate of the CRL4CRBN ubiquitin ligase. Nat. Commun..

[41] Sievers Q.L., Petzold G., Bunker R.D., Renneville A., Słabicki M., Liddicoat B.J., Abdulrahman W., Mikkelsen T., Ebert B.L., Thomä N.H. (2018). Defining the human C2H2 zinc finger degrome targeted by thalidomide analogs through CRBN. Science.

[42] Liu A., Li S., Donnenberg V., Fu J., Gollin S.M., Ma H., Lu C., Stolz N.B., Mapara M.Y., Monaghan S.A. (2018). Immunomodulatory drugs downregulate IKZF1 leading to expansion of hematopoietic progenitors with concomitant block of megakaryocytic maturation. Haematologica.

[43] Pal R., Monaghan S.A., Hassett A.C., Mapara M.Y., Schafer P., Roodman G.D., Ragni M.V., Moscinski L., List A., Lentzsch S. (2010). Immunomodulatory derivatives induce PU.1 down-regulation, myeloid maturation arrest, and neutropenia. Blood.

[44] Rosiñol L., Oriol A., Teruel A.I., Hernández D., López-Jiménez J., de la Rubia J., Granell M., Besalduch J., Palomera L., González Y. (2012). Superiority of bortezomib, thalidomide, and dexamethasone (VTD) as induction pretransplantation therapy in multiple myeloma: A randomized phase 3 PETHEMA/GEM study. Blood.

[45] Moreau P., Attal M., Hulin C., Arnulf B., Belhadj K., Benboubker L., Béné M.C., Broijl A., Caillon H., Caillot D. (2019). Bortezomib, thalidomide, and dexamethasone with or without daratumumab before and after autologous stem-cell transplantation for newly diagnosed multiple myeloma (CASSIOPEIA): A randomised, open-label, phase 3 study. Lancet.

[46] Rosiñol L., Oriol A., Rios R., Sureda A., Blanchard M.J., Hernández M.T., Martínez-Martínez R., Moraleda J.M., Jarque I., Bargay J. (2019). Bortezomib, lenalidomide, and dexamethasone as induction therapy prior to autologous transplant in multiple myeloma. Blood.

[47] Voorhees P.M., Kaufman J.L., Laubach J.P., Sborov D.W., Reeves B., Rodriguez C., Chari A., Silbermann R., Costa L.J., Anderson L.D. (2020). Daratumumab, lenalidomide, bortezomib, and dexamethasone for transplant-eligible newly diagnosed multiple myeloma: The GRIFFIN trial. Blood.

[48] Durie B.G.M., Hoering A., Abidi M.H., Rajkumar S.V., Epstein J., Kahanic S.P., Thakuri M., Reu F., Reynolds C.M., Sexton R. (2017). Bortezomib with lenalidomide and dexamethasone versus lenalidomide and dexamethasone alone in patients with newly diagnosed myeloma without intent for immediate autologous stem-cell transplant (SWOG S0777): A randomised, open-label, phase 3 trial. Lancet.

[49] Kumar S.K., Jacobus S.J., Cohen A.D., Weiss M., Callander N., Singh A.K., Parker T.L., Menter A., Yang X., Parsons B. (2020). Carfilzomib or bortezomib in combination with lenalidomide and dexamethasone for patients with newly diagnosed multiple myeloma without intention for immediate autologous stem-cell transplantation (ENDURANCE): A multicentre, open-label, phase 3, randomised, controlled trial. Lancet Oncol..

[50] Facon T., Kumar S.K., Plesner T., Orlowski R.Z., Moreau P., Bahlis N., Basu S., Nahi H., Hulin C., Quach H. (2021). Daratumumab, lenalidomide, and dexamethasone versus lenalidomide and dexamethasone alone in newly diagnosed multiple myeloma (MAIA): Overall survival results from a randomised, open-label, phase 3 trial. Lancet Oncol..

[51] Callander N.S., Baljevic M., Adekola K., Anderson L.D., Campagnaro E., Castillo J.J., Costello C., Devarakonda S., Elsedawy N., Faiman M. (2022). NCCN Guidelines® Insights: Multiple Myeloma, Version 3.2022. J. Natl. Compr. Cancer Netw..

[52] Dimopoulos M., Spencer A., Attal M., Prince H.M., Harousseau J.-L., Dmoszynska A., Miguel J.S., Hellmann A., Facon T., Foà R. (2007). Lenalidomide plus Dexamethasone for Relapsed or Refractory Multiple Myeloma. N. Engl. J. Med..

[53] Stewart A.K., Rajkumar S.V., Dimopoulos M.A., Masszi T., Špička I., Oriol A., Hájek R., Rosiñol L., Siegel D.S., Mihaylov G.G. (2015). Carfilzomib, Lenalidomide, and Dexamethasone for Relapsed Multiple Myeloma. N. Engl. J. Med..

[54] Siegel D.S., Dimopoulos M.A., Ludwig H., Facon T., Goldschmidt H., Jakubowiak A., Miguel J.S., Obreja M., Blaedel J., Stewart A.K. (2018). Improvement in Overall Survival with Carfilzomib, Lenalidomide, and Dexamethasone in Patients with Relapsed or Refractory Multiple Myeloma. J. Clin. Oncol..

[55] Moreau P., Masszi T., Grzasko N., Bahlis N.J., Hansson M., Pour L., Sandhu I., Ganly P., Baker B.W., Jackson S.R. (2016). Oral Ixazomib, Lenalidomide, and Dexamethasone for Multiple Myeloma. N. Engl. J. Med..

[56] Bahlis N.J., Dimopoulos M.A., White D.J., Benboubker L., Cook G., Leiba M., Ho P.J., Kim K., Takezako N., Moreau P. (2020). Daratumumab plus lenalidomide and dexamethasone in relapsed/refractory multiple myeloma: Extended follow-up of POLLUX, a randomized, open-label, phase 3 study. Leukemia.

[57] Lonial S., Dimopoulos M., Palumbo A., White D., Grosicki S., Spicka I., Walter-Croneck A., Moreau P., Mateos M.V., Magen H. (2015). Elotuzumab Therapy for Relapsed or Refractory Multiple Myeloma. N. Engl. J. Med..

[58] Dimopoulos M.A., Lonial S., White D., Moreau P., Weisel K., San-Miguel J., Shpilberg O., Grosicki S., Špička I., Walter-Croneck A. (2020). Elotuzumab, lenalidomide, and dexamethasone in RRMM: Final overall survival results from the phase 3 randomized ELOQUENT-2 study. Blood Cancer J..

[59] Dimopoulos M., Quach H., Mateos M.-V., Landgren O., Leleu X., Siegel D., Weisel K., Yang H., Klippel Z., Zahlten-Kumeli A. (2020). Carfilzomib, dexamethasone, and daratumumab versus carfilzomib and dexamethasone for patients with relapsed or refractory multiple myeloma (CANDOR): Results from a randomised, multicentre, open-label, phase 3 study. Lancet.

[60] Mateos M.-V., Sonneveld P., Hungria V., Nooka A.K., Estell J.A., Barreto W., Corradini P., Min C.-K., Medvedova E., Weisel K. (2019). Daratumumab, Bortezomib, and Dexamethasone Versus Bortezomib and Dexamethasone in Patients with Previously Treated Multiple Myeloma: Three-year Follow-up of CASTOR. Clin. Lymphoma Myeloma Leuk..

[61] Dimopoulos M.A., Goldschmidt H., Niesvizky R., Joshua D., Chng W.-J., Oriol A., Orlowski R.Z., Ludwig H., Facon T., Hajek R. (2017). Carfilzomib or bortezomib in relapsed or refractory multiple myeloma (ENDEAVOR): An interim overall survival analysis of an open-label, randomised, phase 3 trial. Lancet Oncol..

[62] Dimopoulos M.A., Terpos E., Boccadoro M., Delimpasi S., Beksac M., Katodritou E., Moreau P., Baldini L., Symeonidis A., Bila J. (2021). Daratumumab plus pomalidomide and dexamethasone versus pomalidomide and dexamethasone alone in previously treated multiple myeloma (APOLLO): An open-label, randomised, phase 3 trial. Lancet Oncol..

[63] Richardson P.G., Perrot A., San-Miguel J., Beksac M., Spicka I., Leleu X., Schjesvold F., Moreau P., Dimopoulos M.A., Huang J.S.-Y. (2022). Isatuximab plus pomalidomide and low-dose dexamethasone versus pomalidomide and low-dose dexamethasone in patients with relapsed and refractory multiple myeloma (ICARIA-MM): Follow-up analysis of a randomised, phase 3 study. Lancet Oncol..

[64] Dimopoulos M.A., Dytfeld D., Grosicki S., Moreau P., Takezako N., Hori M., Leleu X., Leblanc R., Suzuki K., Raab M.S. (2018). Elotuzumab plus Pomalidomide and Dexamethasone for Multiple Myeloma. N. Engl. J. Med..

[65] Richardson P.G., Oriol A., Beksac M., Liberati A.M., Galli M., Schjesvold F., Lindsay J., Weisel K., White D., Facon T. (2019). Pomalidomide, bortezomib, and dexamethasone for patients with relapsed or refractory multiple myeloma previously treated with lenalidomide (OPTIMISMM): A randomised, open-label, phase 3 trial. Lancet Oncol..

[66] Shah J.J., Stadtmauer E.A., Abonour R., Cohen A.D., Bensinger W.I., Gasparetto C., Kaufman J.L., Lentzsch S., Vogl D.T., Gomes C.L. (2015). Carfilzomib, pomalidomide, and dexamethasone for relapsed or refractory myeloma. Blood.

[67] Krishnan A., Kapoor P., Palmer J.M., Tsai N.-C., Kumar S., Lonial S., Htut M., Karanes C., Nathwani N., Rosenzweig M. (2018). Phase I/II trial of the oral regimen ixazomib, pomalidomide, and dexamethasone in relapsed/refractory multiple myeloma. Leukemia.

[68] Garderet L., Kuhnowski F., Berge B., Roussel M., Escoffre-Barbe M., Lafon I., Facon T., Leleu X., Karlin L., Perrot A. (2018). Pomalidomide, cyclophosphamide, and dexamethasone for relapsed multiple myeloma. Blood.

[69] Attal M., Lauwers-Cances V., Marit G., Caillot D., Moreau P., Facon T., Stoppa A.M., Hulin C., Benboubker L., Garderet L. (2012). Lenalidomide Maintenance after Stem-Cell Transplantation for Multiple Myeloma. N. Engl. J. Med..

[70] Palumbo A., Cavallo F., Gay F., Di Raimondo F., Ben Yehuda D., Petrucci M.T., Pezzatti S., Caravita T., Cerrato C., Ribakovsky E. (2014). Autologous Transplantation and Maintenance Therapy in Multiple Myeloma. N. Engl. J. Med..

[71] Holstein S.A., Jung S.-H., Richardson P.G., Hofmeister C.C., Hurd D.D., Hassoun H., Giralt S., Stadtmauer E.A., Weisdorf D.J., Vij R. (2017). Updated analysis of CALGB (Alliance) 100104 assessing lenalidomide versus placebo maintenance after single autologous stem-cell transplantation for multiple myeloma: A randomised, double-blind, phase 3 trial. Lancet Haematol..

[72] Jackson G.H., Davies F.E., Pawlyn C., Cairns D.A., Striha A., Collett C., Hockaday A., Jones J.R., Kishore B., Garg M. (2019). Lenalidomide maintenance versus observation for patients with newly diagnosed multiple myeloma (Myeloma XI): A multicentre, open-label, randomised, phase 3 trial. Lancet Oncol..

[73] McCarthy P.L., Holstein S.A., Petrucci M.T., Richardson P.G., Hulin C., Tosi P., Bringhen S., Musto P., Anderson K.C., Caillot D. (2017). Lenalidomide Maintenance After Autologous Stem-Cell Transplantation in Newly Diagnosed Multiple Myeloma: A Meta-Analysis. J. Clin. Oncol..

[74] Jones J.R., Csg O.B.O.T.N.H.-O., Cairns D.A., Gregory W.M., Collett C., Pawlyn C., Sigsworth R., Striha A., Henderson R., Kaiser M.F. (2016). Second malignancies in the context of lenalidomide treatment: An analysis of 2732 myeloma patients enrolled to the Myeloma XI trial. Blood Cancer J..

[75] Chavez B.M., Barnell B.E., Griffith M., Skidmore Z., Griffith O., Tian L., Wartman L.D. (2020). B-Cell Acute Lymphoblastic Leukemia Arising in Patients with a Preexisting Diagnosis of Multiple Myeloma Is a Novel Cancer with High Incidence of TP53 Mutations. Blood.

[76] Germans S.K., Kulak O., Koduru P., Oliver D., Gagan J., Patel P., Anderson L.D., Fuda F.S., Chen W., Jaso J.M. (2020). Lenalidomide-Associated Secondary B-Lymphoblastic Leukemia/Lymphoma—A Unique Entity. Am. J. Clin. Pathol..

[77] Khan S.R., Tariq M., Fayyaz S.M., Soomar S.M., Moosajee M. (2022). Lenalidomide induced secondary Acute Lymphoblastic Leukemia in a Multiple Myeloma patient: A case-report. Leuk. Res. Rep..

[78] Sinit R., Hwang D.G., Vishnu P., Peterson J.F., Aboulafia D.M. (2019). B-cell acute lymphoblastic leukemia in an elderly man with plasma cell myeloma and long-term exposure to thalidomide and lenalidomide: A case report and literature review. BMC Cancer.

[79] Matyskiela M.E., Zhang W., Man H.-W., Muller G., Khambatta G., Baculi F., Hickman M., Lebrun L., Pagarigan B., Carmel G. (2017). A Cereblon Modulator (CC-220) with Improved Degradation of Ikaros and Aiolos. J. Med. Chem..

[80] Thakurta A., Pierceall W.E., Amatangelo M.D., Flynt E., Agarwal A. (2021). Developing next generation immunomodulatory drugs and their combinations in multiple myeloma. Oncotarget.

[81] Hansen J.D., Correa M., Nagy M.A., Alexander M., Plantevin V., Grant V., Whitefield B., Huang D., Kercher T., Harris R. (2020). Discovery of CRBN E3 Ligase Modulator CC-92480 for the Treatment of Relapsed and Refractory Multiple Myeloma. J. Med. Chem..

[82] Matyskiela M.E., Lu G., Ito T., Pagarigan B., Lu C.-C., Miller K., Fang W., Wang N.-Y., Nguyen D., Houston J. (2016). A novel cereblon modulator recruits GSPT1 to the CRL4CRBN ubiquitin ligase. Nature.

[83] Hao B.-B., Li X.-J., Jia X.-L., Wang Y.-X., Zhai L.-H., Li D.-Z., Liu J., Zhang D., Chen Y.-L., Xu Y.-H. (2020). The novel cereblon modulator CC-885 inhibits mitophagy via selective degradation of BNIP3L. Acta Pharmacol. Sin..

[84] Zhao M., Hu M., Chen Y., Liu H., Chen Y., Liu B., Fang B. (2021). Cereblon modulator CC-885 induces CRBN-dependent ubiquitination and degradation of CDK4 in multiple myeloma. Biochem. Biophys. Res. Commun..

[85] Bjorklund C.C., Kang J., Lu L., Amatangelo M., Chiu H., Hagner P., Gandhi A.K., Pourdehnad M., Klippel A., Thakurta A. (2016). CC-122 Is a Cereblon Modulating Agent That Is Active in Lenalidomide-Resistant and Lenalidomide/Dexamethasone-Double-Resistant Multiple Myeloma Pre-Clinical Models. Blood.

[86] Bjorklund C.C., Kang J., Amatangelo M., Polonskaia A., Katz M., Chiu H., Couto S., Wang M., Ren Y., Ortiz M. (2019). Iberdomide (CC-220) is a potent cereblon E3 ligase modulator with antitumor and immunostimulatory activities in lenalidomide- and pomalidomide-resistant multiple myeloma cells with dysregulated CRBN. Leukemia.

[87] Renneville A., Gasser J.A., Grinshpun D.E., Beltran P.M.J., Udeshi N.D., Matyskiela M.E., Clayton T., McConkey M., Viswanathan K., Tepper A. (2021). Avadomide Induces Degradation of ZMYM2 Fusion Oncoproteins in Hematologic Malignancies. Blood Cancer Discov..

[88] Richardson P.G., Ocio E., Raje N.S., Gregory T., White D., Oriol A., Sandhu I., Raab M.-S., LeBlanc R., Rodriguez C. (2021). CC-92480, a Potent, Novel Cereblon E3 Ligase Modulator (CELMoD) Agent, in Combination with Dexamethasone (DEX) and Bortezomib (BORT) in Patients (pts) with Relapsed/Refractory Multiple Myeloma (RRMM): Preliminary Results from the Phase 1/2 Study CC-92480-MM-002. Blood.

[89] Richardson P.G., Vangsted A.J., Ramasamy K., Trudel S., Martínez J., Mateos M.-V., Rodríguez Otero P., Lonial S., Popat R., Oriol A. (2020). First-in-human phase I study of the novel CELMoD agent CC-92480 combined with dexamethasone (DEX) in patients (pts) with relapsed/refractory multiple myeloma (RRMM). J. Clin. Oncol..

[90] van de Donk N.W., Popat R., Larsen J., Minnema M.C., Jagannath S., Oriol A., Zonder J., Richardson P.G., Rodriguez-Otero P., Badros A.Z. (2020). First Results of Iberdomide (IBER.;CC-220) in Combination with Dexamethasone (DEX) and Daratumumab (DARA) or Bortezomib (BORT) in Patients with Relapsed/Refractory Multiple Myeloma (RRMM). Blood.

[91] van de Donk N.W.C.J., Popat R., Hulin C., Jagannath S., Oriol A., Richardson P.G., Facon T., Weisel K., Larsen J.T., Minnema M. (2022). P07: RESULTS FROM THE CC-220-MM-001 DOSE-EXPANSION PHASE OF IBERDOMIDE PLUS DEXAMETHASONE IN PATIENTS WITH RELAPSED/REFRACTORY MULTIPLE MYELOMA. HemaSphere.

[92] Rasco D.W., Papadopoulos K.P., Pourdehnad M., Gandhi A.K., Hagner P.R., Li Y., Wei X., Chopra R., Hege K., DiMartino J.F. (2019). A First-in-Human Study of Novel Cereblon Modulator Avadomide (CC-122) in Advanced Malignancies. Clin. Cancer Res..

[93] Nastoupil L.J., Kuruvilla J., Chavez J.C., Bijou F., Witzig T.E., Santoro A., Flinn I.W., Boccomini C., Kenkre V.P., Corradini P. (2022). Phase Ib study of avadomide (CC-122) in combination with rituximab in patients with relapsed/refractory diffuse large B-cell lymphoma and follicular lymphoma. eJHaem.

[94] Michot J.-M., Bouabdallah R., Vitolo U., Doorduijn J.K., Salles G., Chiappella A., Zinzani P.L., Bijou F., Kersten M.J., Sarmiento R. (2020). Avadomide plus obinutuzumab in patients with relapsed or refractory B-cell non-Hodgkin lymphoma (CC-122-NHL-001): A multicentre, dose escalation and expansion phase 1 study. Lancet Haematol..

[95] Li L., Xue W., Shen Z., Liu J., Hu M., Cheng Z., Wang Y., Chen Y., Chang H., Liu Y. (2020). A Cereblon Modulator CC-885 Induces CRBN- and p97-Dependent PLK1 Degradation and Synergizes with Volasertib to Suppress Lung Cancer. Mol. Ther. Oncolytics.

[96] Winter G.E., Buckley D.L., Paulk J., Roberts J.M., Souza A., Dhe-Paganon S., Bradner J.E. (2015). Phthalimide conjugation as a strategy for in vivo target protein degradation. Science.

[97] Zhang X., Lee H.C., Shirazi F., Baladandayuthapani V., Lin H., Kuiatse I., Wang H., Jones R.J., Berkova Z., Singh R.K. (2018). Protein targeting chimeric molecules specific for bromodomain and extra-terminal motif family proteins are active against pre-clinical models of multiple myeloma. Leukemia.

[98] Lim S.L., Damnernsawad A., Shyamsunder P., Chng W.J., Han B.C., Xu L., Pan J., Pravin D.P., Alkan S., Tyner J.W. (2019). Proteolysis targeting chimeric molecules as therapy for multiple myeloma: Efficacy, biomarker and drug combinations. Haematologica.

[99] Steinebach C., Ng Y.L.D., Sosič I., Lee C.-S., Chen S., Lindner S., Vu L.P., Bricelj A., Haschemi R., Monschke M. (2020). Systematic exploration of different E3 ubiquitin ligases: An approach towards potent and selective CDK6 degraders. Chem. Sci..

[100] Ng Y.L.D., Ramberger E., Bohl S.R., Dolnik A., Steinebach C., Conrad T., Müller S., Popp O., Kull M., Haji M. (2022). Proteomic profiling reveals CDK6 upregulation as a targetable resistance mechanism for lenalidomide in multiple myeloma. Nat. Commun..

[101] Papatzimas J.W., Gorobets E., Maity R., Muniyat M.I., Maccallum J.L., Neri P., Bahlis N.J., Derksen D.J. (2019). From Inhibition to Degradation: Targeting the Antiapoptotic Protein Myeloid Cell Leukemia 1 (MCL1). J. Med. Chem..

[102] Medvar B., Raghuram V., Pisitkun T., Sarkar A., Knepper M.A. (2016). Comprehensive database of human E3 ubiquitin ligases: Application to aquaporin-2 regulation. Physiol. Genom..

[103] Shirasaki R., Matthews G.M., Gandolfi S., Simoes R.D.M., Buckley D.L., Vora J.R., Sievers Q.L., Brüggenthies J.B., Dashevsky O., Poarch H. (2021). Functional Genomics Identify Distinct and Overlapping Genes Mediating Resistance to Different Classes of Heterobifunctional Degraders of Oncoproteins. Cell Rep..

[104] He Y., Khan S., Huo Z., Lv D., Zhang X., Liu X., Yuan Y., Hromas R., Xu M., Zheng G. (2020). Proteolysis targeting chimeras (PROTACs) are emerging therapeutics for hematologic malignancies. J. Hematol. Oncol..

[105] Qi S.-M., Dong J., Xu Z.-Y., Cheng X.-D., Zhang W.-D., Qin J.-J. (2021). PROTAC: An Effective Targeted Protein Degradation Strategy for Cancer Therapy. Front. Pharmacol..

[106] Li X., Pu W., Zheng Q., Ai M., Chen S., Peng Y. (2022). Proteolysis-targeting chimeras (PROTACs) in cancer therapy. Mol. Cancer.

[107] Gao X., Burris H.A., Vuky J., Dreicer R., Sartor A.O., Sternberg C.N., Percent I.J., Hussain M.H.A., Kalebasty A.R., Shen J. (2022). Phase 1/2 study of ARV-110, an androgen receptor (AR) PROTAC degrader, in metastatic castration-resistant prostate cancer (mCRPC). J. Clin. Oncol..

[108] Hamilton E., Vahdat L., Han H.S., Ranciato J., Gedrich R., Keung C.F., Chirnomas D., Hurvitz S. (2022). Abstract PD13-08: First-in-human safety and activity of ARV-471, a novel PROTAC® estrogen receptor degrader, in ER+/HER2- locally advanced or metastatic breast cancer. Cancer Res..

[109] Starodub A., Gollerkeri A., De Savi C., Dey J., Agarwal S., Donohue S., Perea R., Klaus C., Gollob J. (2022). Phase 1 study of KT-333, a targeted protein degrader, in patients with relapsed or refractory lymphomas, large granular lymphocytic leukemia, and solid tumors. J. Clin. Oncol..

